# A Musical Approach to Speech Melody

**DOI:** 10.3389/fpsyg.2018.00247

**Published:** 2018-03-05

**Authors:** Ivan Chow, Steven Brown

**Affiliations:** Department of Psychology, Neuroscience & Behaviour, McMaster University, Hamilton, ON, Canada

**Keywords:** speech melody, speech prosody, music, phonetics, phonology, language

## Abstract

We present here a musical approach to speech melody, one that takes advantage of the intervallic precision made possible with musical notation. Current phonetic and phonological approaches to speech melody either assign localized pitch targets that impoverish the acoustic details of the pitch contours and/or merely highlight a few salient points of pitch change, ignoring all the rest of the syllables. We present here an alternative model using musical notation, which has the advantage of representing the pitch of *all* syllables in a sentence as well as permitting a specification of the intervallic excursions among syllables and the potential for group averaging of pitch use across speakers. We tested the validity of this approach by recording native speakers of Canadian English reading unfamiliar test items aloud, spanning from single words to full sentences containing multiple intonational phrases. The fundamental-frequency trajectories of the recorded items were converted from hertz into semitones, averaged across speakers, and transcribed into musical scores of relative pitch. Doing so allowed us to quantify local and global pitch-changes associated with declarative, imperative, and interrogative sentences, and to explore the melodic dynamics of these sentence types. Our basic observation is that speech is atonal. The use of a musical score ultimately has the potential to combine speech rhythm and melody into a unified representation of speech prosody, an important analytical feature that is not found in any current linguistic approach to prosody.

## Introduction

It is common to refer to the pitch properties of speech as “speech melody” in the study of prosody (Bolinger, 1989; Nooteboom, 1997; Ladd, 2008). However, is this simply a metaphorical allusion to musical melodies, or does speech actually have a similar system of pitch relations as music? If it does not, what is the nature of speech’s melodic system compared to that of music? A first step toward addressing such questions is to look at speech and music using the same analytical tools and to examine speech as a true melodic system comprised of pitches (tones) and intervals. This is the approach that we aim to implement and test in the present study. In fact, it was the approach that was adopted in the first theoretical treatise about English intonation, namely Joshua Steele’s *An Essay Toward Establishing the Melody and Measure of Speech to be Expressed and Perpetuated by Peculiar Symbols*, published in 1775. Steele laid out a detailed musical model of both the melody and rhythm of speech (we will only concern ourselves with the melodic concepts here). He represented syllabic pitch as a relative-pitch system using a musical staff and a series of “peculiar symbols” that would represent the relative pitch and relative duration of each spoken syllable of an utterance. The key innovation of Steele’s approach from our standpoint is that he attempted to represent the pitches of *all* of the syllables in the sentences that he analyzed. Another advantage of his approach is that his use of the musical score allowed for both the rhythm and melody of speech to be analyzed, both independently of one another and interactively.

This is in stark contrast to most contemporary approaches to speech melody in linguistics that highlight a subset of salient syllabic pitches and thereby ignore all the rest of the melodic signal in a sentence, assuming a process of interpolation between those salient pitches. Many such approaches are based on qualitative labeling of pitch transitions, rather than acoustic quantification of actual pitch changes occurring in an utterance. At present, no musical elements are incorporated into any of the dominant phonetic or phonological models of speech melody. These models include autosegmental metrical (AM) theory (Bruce, 1977; Pierrehumbert, 1980; Beckman and Pierrehumbert, 1986; Gussenhoven, 2004; Ladd, 2008), the command-response (CR) model (Fujisaki and Hirose, 1984; Fujisaki et al., 1998; Fujisaki and Gu, 2006), and the “parallel encoding and target approximation” model (Xu, 2005; Prom-on et al., 2009). Perhaps the closest approximation to a musical representation is Mertens’ (2004) Prosogram software, which automatically transcribes speech melody and rhythm into a series of level and contoured tones (see also Mertens and d’Alessandro, 1995; Hermes, 2006; Patel, 2008). Prosogram displays pitch measurements for each syllable by means of a level, rising, or falling contour, where the length of each contour represents syllabic duration (Mertens, 2004). However, this seems to be mainly a transcription tool, rather than a theoretical model for describing the melodic dynamics of speech.

### Prosody vs. Speech Melody vs. Intonation

Before comparing the three dominant models of speech melody with the musical approach that we are proposing (see next section), we would like to first define the important terms “prosody,” “speech melody,” and “intonation,” and discuss how they relate to one another, since these terms are erroneously taken to be synonymous. “Prosody” is an umbrella term that refers to variations in all suprasegmental parameters of speech, including pitch, but also duration and intensity. On the other hand, “speech melody” and “intonation” refer strictly to the *pitch* changes associated with speech communication, where “intonation” is a more restrictive term than “speech melody”. “Speech melody” refers to the pitch trajectory associated with utterances of any length. This term does not entail a distinction as to whether pitch is generated lexically (tone) or post-lexically (intonation), or whether the trajectory (or a part thereof) serves a linguistic or paralinguistic function.

While “speech melody” refers to all pitch variations associated with speech communication, “intonation” refers specifically to the pitch contour of an utterance generated *post-lexically* and that is associated with the concept of an “intonational phrase” (Ladd, 2008). Ladd (2008) defines intonation as a *linguistic* term that involves categorical discrete-to-gradient correlations between pattern and meaning. Intonation differs from pitch changes associated with “tones” or “accents”, which are determined lexically and which are associated with the syllable. By contrast, *paralinguistic* meanings (e.g., emotions and emphatic force) involve continuous-to-gradient correlations (Ladd, 2008). For example, the angrier someone is, the wider is the pitch range and intensity range of their speech (Fairbanks and Pronovost, 1939; Murray and Arnott, 1993).

### Contemporary Phonological Models of Speech Melody

In this section, we review three dominant models of speech melody: AM theory, the CR model, and the parallel encoding and target approximation (PENTA) model. Briefly, AM theory only highlights phonologically salient melodic excursions associated with key elements in intonational phrases, including pitch accents and boundary tones (Pierrehumbert, 1980; Liberman and Pierrehumbert, 1984). On the other hand, CR imitates speech melody by mathematically generating pitch contours, and connecting pitch targets so as to create peaks and valleys along a gradually declining line (Cohen et al., 1982; Fujisaki, 1983). Finally, PENTA assigns a pitch target to each and every syllable of an intonational phrase. Each target is mathematically derived from a number of factors, including lexical stress, narrow focus, modality, and position of the syllable within an intonational phrase. The final pitch contour is then generated as an approximation of the original series of pitch targets, in which distance between pitch targets is reduced due to contextual variations (Xu, 2005, 2011).

#### Auto-Segmental Metrical Theory

The ToBI (Tone and Break Index) system of prosodic notation builds on assumptions made by AM theory (Pierrehumbert, 1980; Beckman and Ayers, 1997). Phonologically salient prosodic events are marked by pitch accents (represented in ToBI as H^∗^, where H means high) at the beginning and middle of an utterance; the end is marked by a boundary tone (L–L%, where L means low); and the melodic contour of the entire utterance is formed by interpolation between pitch accents and the boundary tone. Under this paradigm, pitch accents serve to mark local prosodic events, including topic word, narrow focus, and lexical stress. Utterance-final boundary tones serve to convey modality (i.e., question vs. statement; continuity vs. finality). Pitch accents and boundary tones are aligned with designated stressed syllables in the utterance and are marked with a high (H) or low (L) level tone. In addition, pitch accents and boundary tones can further combine with a series of H and L tones to convey different modalities, as well as other subtle nuances in information structure (Hirschberg and Ward, 1995; Petrone and Niebuhr, 2014; German and D’Imperio, 2016; Féry, 2017). Consequently, the melodic contour of an utterance is defined by connecting pitch accents and boundary tones, whereas strings of syllables between pitch accents are unspecified with regard to tone and are treated as transitions. AM is considered to be a “compositional” method that looks at prosody as a generative and combinatorial system whose elements consist of the abovementioned tone types. This compositionality might suggest a mechanistic similarity to music, with its combinatorial system of scaled pitches. However, the analogy does not ultimately work, in large part because the tones of ToBI analyses are highly underspecified at the pitch level; the directionality of pitch movement is marked, but not the magnitude of the change.

#### Command-Response Model

Fujisaki and Hirose (1984) and Fujisaki and Gu (2006) proposed the CR model based on the physiological responses of the human vocal organ. In this model, declination is treated as the backbone of the melodic contour (Cohen et al., 1982; Fujisaki, 1983). Declination is a reflection of the physiological conditions of phonation: speech occurs during exhalation. As the volume of air in the lungs decreases, the amount of air passing through the larynx also decreases, as does the driving force for vocalization, namely subglottal pressure. This results in a decrease in the frequency of vocal-fold vibration. CR replicates this frequency change by way of a gradual melodic downtrend as the utterance progresses. In this model, the pitch range of the melodic contour is defined by a topline and a baseline. Both lines decline as the utterance progresses, although the topline declines slightly more rapidly than the baseline, making the overall pitch range gradually narrower (i.e., more compressed) over time. In addition to declination, tone commands introduce localized peaks and valleys along the global downtrend. Although tone commands do not directly specify the target pitch of the local peaks and valleys, they are expressed as mathematical functions that serve to indicate the strength and directionality of these localized pitch excursions. Both AM and CR are similar in that pitch contours are delineated by sparse tonal specifications, and that syllables between tone targets are treated as transitions whose pitches are unspecified. However, the two models differ in that tone commands in the CR model are not necessarily motivated by phonologically salient communicative or linguistic functions. These commands are only used to account for pitch variations in order to replicate the observed pitch contours. This difference thus renders the CR model largely descriptive (phonetic), rather than interpretive (phonological), as compared with AM theory.

#### Parallel Encoding and Target Approximation Model

PENTA (Xu, 2005; Prom-on et al., 2009) takes an articulatory-functional approach to representing speech melody. It aims to explain how speech melody works as a system of communication. Recognizing the fact that different communicative functions are simultaneously conveyed by the articulatory system, PENTA begins with a list of these functions and encodes them in a parallel manner. Each syllable obligatorily carries a tone target. The resulting melodic movement for each syllable is generated as an approximation of a level or dynamic tone-target. The pitch target of each syllable is derived based on its inherent constituent communicative functions that coexist in parallel (e.g., lexical, sentential, and focal). Pitch targets are then implemented in terms of contextual distance, pitch range, strength, and duration. The implementation of each pitch target is said to be approximate, as pitch movements are subject to contextual variations. According to Xu and Xu (2005), the encoding process can be universal or language-specific. In addition, this process can vary due to interference between multiple communicative functions when it comes to the rendering of the eventual melodic contour. In other words, how well the resulting contour resembles the target depends on factors such as contextual variation (anticipatory or carry-over, assimilatory or dissimilatory) and articulatory effort. PENTA is similar to the CR model in that the fundamental frequency (*F*_0_) trajectory of an utterance is plotted as “targets” based on a number of parameters. Such parameters include directionality of the pitch changes, slope of the pitch target, and the speed at which a pitch target is approached. Nonetheless, PENTA sets itself apart from CR and AM in that it establishes a tone target for *every* syllable, whereas CR and AM only assign pitch accents/targets to syllables associated with localized phonologically-salient events (e.g., pitch accents, boundary tones).

Perhaps the only contemporary system that combines rhythm and melody in the same analysis is Rhythm and Pitch, or RaP (Dilley and Brown, 2005; Breen et al., 2012). While based largely on AM’s system of H’s and L’s to represent tones, Breen et al. (2012, p. 277) claim that RaP differs from ToBI in that it “takes into account developments in phonetics, phonology and speech technology since the development of the original ToBI system.” Instead of using numbers to represent relative boundary strength on the “Breaks” tier in ToBI, RaP uses “X” and “x” to mark three degrees of prominence (strong beat, weak beat, and no beat), as well as “))” and “)” to mark two degrees of boundary strength. On the “rhythm” tier, strong beats are assigned to lexically stressed syllables based on metrical phonology (Nespor and Vogel, 1986; Nespor and Vogel, 2007). In addition, the assignment of prominence follows the “obligatory contour principle” (Leben, 1973; Yip, 1988) by imposing that prominent syllables must be separated from one another by at least one non-prominent syllable, as well as by differences in the phonetic realization of content vs. function words. Although RaP sets itself apart from other systems by acknowledging the existence of rhythm and beats (i.e., pockets of isochronous units in lengthy syllable strings) as perceptual parameters, it still treats rhythm and pitch as completely separate, rather than integrated, parameters, and makes no provision to analyze or account for potential interactions between syllabic duration and pitch.

### Toward a Musical Approach

While all of the linguistic models discussed here claim to represent speech prosody, the fact that speech rhythm is integral to speech prosody and that rhythm and melody interact is largely ignored. As such, these models are only successful at representing some aspects of speech prosody, but present limitations at capturing the larger picture. The use of musical notation to represent speech prosody offers several advantages over AM theory and PENTA. First, the use of the semitone-based chromatic scale provides for a more precise characterization of speech melody, compared to the impoverished system of acoustically unspecified H and L tones found in ToBI transcriptions. As pointed out by Xu and Xu (2005), AM Theory is strictly a linear model in that the marking of one tone as H or L essentially depends on the pitch of its adjacent syllables (tones). It is hence impossible to analyze speech melody beyond the scope of three syllables under the AM paradigm. In addition, the use of semitones might in fact provide a superior approach to describing speech melody than plotting pitch movements in hertz, since semitones correspond to the logarithmic manner by which pitches (and by extension intervals) are perceived by the human ear, although the auditory system clearly has a much finer pitch-discrimination accuracy than the semitone (Oxenham, 2013). In addition, musical notation can simultaneously represent both the rhythm and melody of speech using a common set of symbols, which is a feature that no current linguistic model of speech prosody can aspire to. As such, the use of musical notation not only provides a new and improved paradigm for model speech melody in terms of intervals, but it also provides a more precise and user-friendly approach that can be readily integrated into current prosody research to further our understanding of the correspondence between prosodic patterns and their communicative functions. Speech melody denoted by musical scores can be readily learned and replicated by anyone trained in reading such scores. As a result, transcribing speech prosody with musical notation could ultimately serve as an effective teaching tool for learning the intonation of a foreign language.

Finally, with regard to the dichotomy in linguistics between “phonetics” and “phonology” (Pierrehumbert, 1999), we believe that the use of musical notation to represent speech melody should be first and foremost tested as a *phonetic* system guided by the amount of acoustic detail present in the observed melodic contours. These details presumably serve to express both linguistic and paralinguistic functions. To further understand the communicative functions of speech melody, the correspondence between specific prosodic patterns and their meaning would then fall under the category of *phonological* research, using the musical approach as a research tool. For example, the British school of prosodic phonology has historically taken a compositional approach to representing speech melody and its meaning, where melody is comprised of tone-units. Each tone-unit contains one of six possible tones (Halliday, 1967, 1970; O’Connor and Arnold, 1973; among others) – high-level, low-level, rise, fall, rise-fall and fall-rise – each of which conveys a specific type of pragmatic information. For example, the fall-rise often suggests uncertainty or hesitation, whereas the rise-fall often indicates that the speaker is surprised or impressed. The length of a tone-unit spans from a single word to a complete sentence. The “tonic syllable” is the essential part of the tone-unit that carries one of the six abovementioned tones. Stressed syllables preceding the tonic are referred to as “heads”; unstressed syllables preceding the head are referred to as “pre-heads.” Finally, unstressed syllables following the tonic are referred to as the “tail.”

The principal aim of the current study is to examine the utility of using a musical approach to speech melody and to visualize the results quantitatively as plots of relative pitch using musical notation. In this vocal-production study, we had 19 native speakers of Canadian English read aloud a series of 19 test items, spanning from single words to full sentences containing multiple intonational phrases. These sentences were designed to examine declination, modality, narrow focus, and utterance-final boundary tones. We decided to analyze these particular features because their correspondence to linguistic meaning is relatively well-defined and because their implementation is context-independent. In other words, melodic patterns associated with the test sentences remain stable when placed within various hypothetical social contexts (Grice and Baumann, 2007; Prieto, 2015). We transcribed participants’ melodic contours into relative-pitch representations down to the level of the semitone using musical notation. The aim was to provide a detailed quantitative analysis of the relative-pitch properties of the test items, demonstrate mechanistic features of sentence melody (such as declination, pitch accents, and boundary effects), and highlight the utility of the method for averaging productions across multiple speakers and visualizing the results on a musical staff. In doing so, this analysis would help revive the long-forgotten work of Steele (1775) and his integrative method of representing both speech rhythm and melody using a common system of musical notation. A companion musical model of speech rhythm using musical notation is presented elsewhere (Brown et al., 2017).

## Materials and Methods

### Participants

Nineteen participants (16 females, mean age 19.8) were recruited from the introductory psychology mass-testing pool at McMaster University. Eighteen of them were paid a nominal sum for their participation, while one was given course credit. All of them were native speakers of Canadian English. Two thirds of the participants had school training or family experience in a second language. Participants gave written informed consent for taking part of the study, which was approved by the McMaster Research Ethics Board.

### Test Corpus

Participants were asked to read a test corpus of 19 test items ranging from single words to various types of sentences, as shown in **Table 1**. This corpus included declarative sentences, interrogatives, an imperative, and sentences with narrow focus specified at different locations. The purpose of using this broad spectrum of sentences was to analyze different prosodic patterns in order to construct a new model of speech melody based on a syllable-by-syllable analysis of pitch.

**Table 1:** Sentences in the test corpus.

**Concatenation: Noun → Phrase → Sentence**
Yellow
Telephone
The yellow telephone
The yellow telephone rang.
The yellow telephone rang frequently.

Saturday
Morning
Saturday morning

On Saturday morning, the yellow telephone rang.
Alanna
Alanna picked it up.
The yellow telephone rang until Alanna picked it up.

**Narrow Focus**
MY roommate had three telephones.
My ROOMMATE had three telephones.
My roommate had THREE telephones.
My roommate had three TELEPHONES.

**Imperative**
Telephone my house!

**Interrogatives**
Whose telephone is that? (WH-question)
Is that my telephone? (yes–no question)


In addition to examining the melody of full sentences, we used a building-block approach that we call a “concatenation” technique in order to observe the differences in the pitch contours of utterances between (1) citation form (i.e., a single word all on its own), (2) phrase form, and (3) a full sentence, which correspond, respectively, to the levels of prosodic word, intermediate phrase, and intonational phrase in the standard phonological hierarchy (Nespor and Vogel, 1986). For example, the use of the concatenation technique resulted in the generation of corpus items that spanned from the single words “Yellow” and “Telephone,” to the adjectival phrase “The yellow telephone,” to the complete sentences “The yellow telephone rang” and “The yellow telephone rang frequently.” This allowed us to compare the tune of “yellow” in citation form to that in phrases and sentences. Gradually increasing the length of the sentences allowed us to observe the corresponding pitch changes for all the words in the sentences.

### Procedure

Before the experiment began, participants filled out questionnaires. They were then brought into a sound-attenuated booth and seated in front of a computer screen. Test sentences were displayed using Presentation^®^ software (Neurobehavioral Systems, Albany, CA, United States). All vocal recordings were made using a Sennheiser tabletop microphone, and recorded at a 44.1 kHz sampling rate as 16 bit depth WAV files on Presentation’s internal recording system. Before the test sentences were read, warm-up tasks were performed in order to assess the participant’s vocal range and habitual vocal pitch. This included throat clears, coughs, sweeps to the highest and lowest pitches, and the reading of the standard “Grandfather” passage.

Participants were next shown the items of the test corpus on a computer screen and were asked to read them aloud in an emotionally neutral manner as if they were engaging in a casual conversation. The 19 items were presented in a different random order for each participant. Each item was displayed on the screen for a practice period of 10 s during which the participant could practice saying it out loud. After this, a 10 s recording period began as the participant was asked to produce the utterance fluently twice without error. The second one was analyzed. In the event of a speech error, participants were instructed to simply repeat the item. For words that were placed under narrow focus, the stressed word or syllable was written in capital letters (e.g., “My ROOMmate had three telephones”).

### Analysis

In order to transcribe the pitch contour of the recorded speech, we analyzed the *F*_0_ trajectory of the digitized speech signal using Praat (Boersma and Weenink, 2015), an open-source program for the acoustic analysis of speech. Steady-state parts of the voiced portion of each syllable were manually delineated – including the vowel and preceding voiced consonants – and the average pitch (in Hz) was extracted. This was done manually for all 2,337 syllables (123 syllables × 19 participants) in the dataset. In a number of situations, the terminal pitch of a test item was spoken in creaky voice such that a reliable pitch measurement was not obtainable for that syllable. When this occurred, it affected either the last syllable of a single word spoken in citation form or the last syllable of the final word of a multi-word utterance. In both cases, it was necessary to discard the entire item from the dataset. While the preceding syllabic pitches could be estimated with accuracy, the absence of the last syllable would mean that the last interval in the group analysis would be inaccurate if the other syllables were included. For this reason, the full item was discarded. This affected 13% of the 361 test items (19 items × 19 participants), almost all of them terminal syllables spoken in creaky voice for which no reliable pitch measurement could be obtained.

Pitch-changes (intervals) were converted from Hz into “cents change” using the participant’s habitual pitch as the reference for the conversion, where 100 cents is equal to one equal-tempered semitone in music. Conversion from Hz to semitones allows for a comparison of intervals across gender and age (Whalen and Levitt, 1995), as well as for group averaging of production. In order to get an estimate of a participant’s habitual pitch, we took the mean frequency of the productions of all the items in the test corpus, excluding entire items that were discarded due to creaky voice. Musical intervals were assigned after the group averaging had been completed. Intervals were assigned to the nearest semitone, assuming the 12-tone chromatic scale, where a ±50-cent criterion separated adjacent chromatic pitches. It is important to note that our transcriptions are no more accurate than the semitone level and that we did not attempt to capture microtonality in the speech signal. Hence, it sufficed for us to assign an interval to the closest reasonable semitone. For example, a major second, which is a 200 cent interval, was defined by pitch transitions occurring anywhere in the span from 150 to 249 cents. It is also important to note that “quantization” to the nearest interval was only ever done with the group data, and that all single-subject data were kept in their raw form in cents throughout all analyses. For the full-corpus analysis of intervals that will be presented in **Figure 8**, intervals are shown in raw form without any rounding to semitone categories.

**FIGURE 1:**
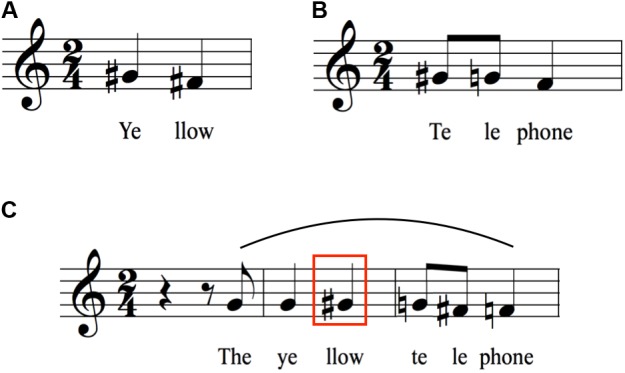
Concatenation of “yellow” and “telephone” to form “The yellow telephone.” The figure shows the average relative-pitch changes associated with the syllables of the test items. For **Figures 1**–**7**, the transcriptions are shown using a treble clef, with the habitual pitch arbitrarily assigned to middle G. All intervals are measured in semitones with reference to participants’ habitual pitch, down to the nearest semitone, as based on a 50-cent pitch window around the interval. **(A)** Citation form of “yellow.” **(B)** Citation form of “telephone.” **(C)** Concatenation to form the adjectival phrase “The yellow telephone.” Notice the contour reversal for “llow” (red box) compared to citation form in **(A)**. The curved line above the staff indicates a melodic arch pattern in **Figures 1**–**7**.

**FIGURE 2:**
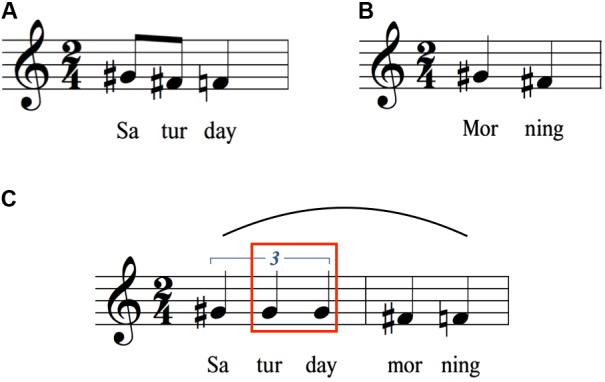
Concatenation of “Saturday” and “morning” to form “Saturday morning”. **(A)** Citation form of “Saturday.” **(B)** Citation form of “morning.” **(C)** Concatenation to form the phrase “Saturday morning.” Notice the contour reversal for “tur” and “day” (red box) compared to the citation form in **(A)**.

**FIGURE 3:**
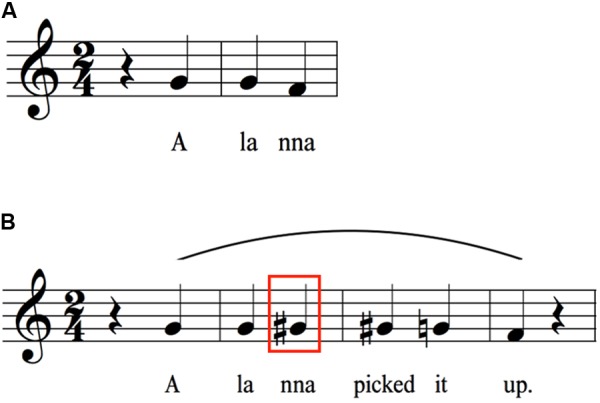
Expansion of “Alanna” to “Alanna picked it up.” **(A)** Citation form of “Alanna.” **(B)** Expansion to create the sentence “Alanna picked it up.” Notice the contour reversal for “nna” (red box) compared to the citation form in **(A)**.

**FIGURE 4:**
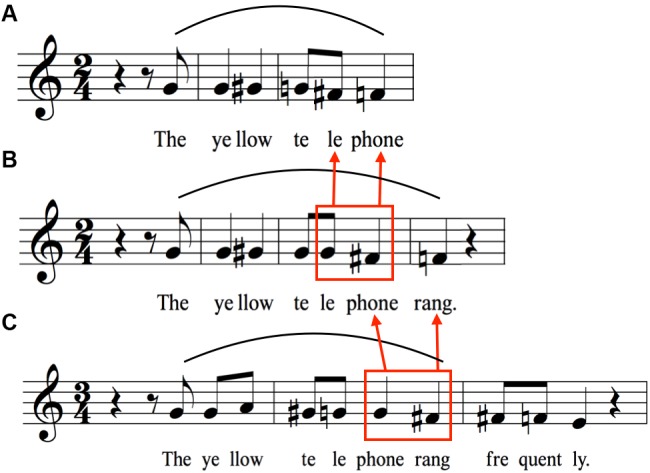
Expansion to from longer sentences starting with “The yellow telephone”. As words are added to the end of the sentence, declination is suspended on the last syllable of the previous sentence such that the characteristic drop between the penultimate and final syllables can serve to mark the end of the sentence at the newly added final word. **(A)** Melodic pattern for “The yellow telephone rang.” **(B)** Melodic pattern generated by adding the word “rang” to the end of the sentence in **(A)**. The red box highlights the difference in pitch height between the syllables “le-phone” in **(A,B)**, demonstrating the suspension of declination occurring on these syllables in **(B)**. **(C)** Melodic pattern generated by adding the word “frequently” to the end of the sentence in **(B)**. The red box around “phone rang” highlights the difference in pitch height between these syllables in **(B,C)**. The point of suspension of declination has moved from “phone” to “rang” in **(C)**.

**FIGURE 5:**
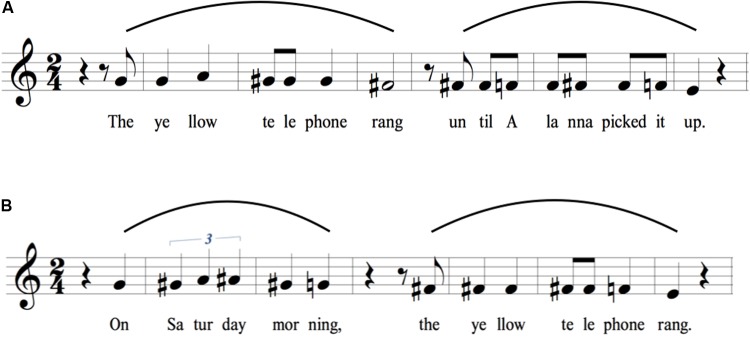
Melodic contours for long sentences consisting of two intonational phrases, characterized by two melodic arches. Both sentences in this figure contain phrases based on items found in **Figures 2**–**4**. The sentence in **(A)** combines sentence B in **Figure 4** and sentence B in **Figure 3**. The sentence in **(B)** combines sentence C in **Figure 2** and sentence B in **Figure 4**. The melodic contour of a long sentence consisting of two intermediate phrases shows two arched patterns, similar to those in the sentences presented in **Figure 4**. These sentences provide further evidence of contour reversals, melodic arches, suspensions of declination, and terminal drops. See text for details.

**FIGURE 6:**
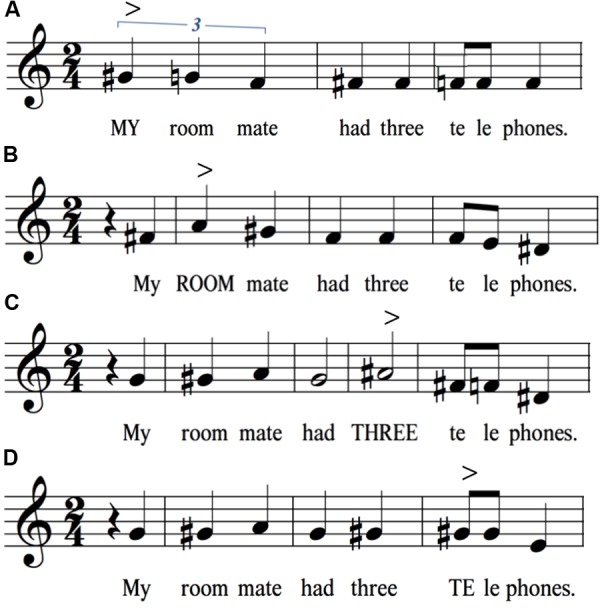
Identical sentences but with narrow focus placed sequentially on different words. All four panels have the same string of words, but with narrow focus placed on either **(A)** my, **(B)** roommate, **(C)** three, or **(D)** telephone. Pitch rises are observed on the focus word in all instances but the last one. The symbol “>” signifies a point of focus or accent. For ease of presentation, only the stressed syllable of roommate and telephone is shown in block letters in the transcription.

**FIGURE 7:**
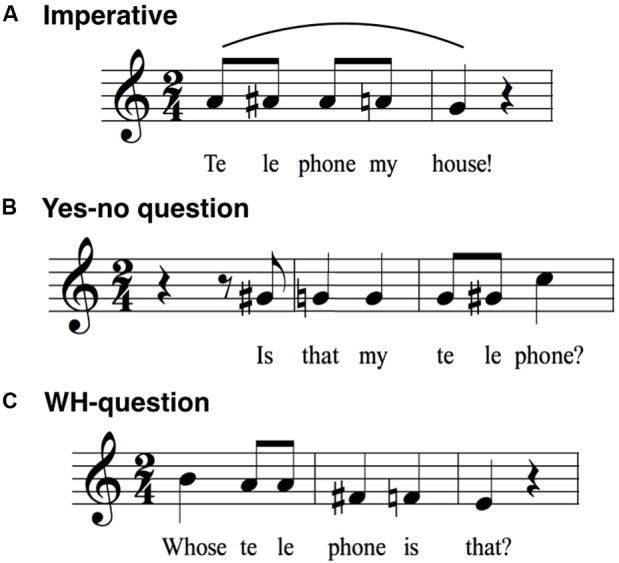
Sentence modality. A comparison is made between **(A)** an imperative sentence, **(B)** a yes–no interrogative, and **(C)** a WH-interrogative. Of interest here is the absence of declination for the imperative statement in **(A)**, as well as the large terminal rise at the end of the yes–no interrogative in **(B)**.

As a normalization procedure for the group results, the intervals were averaged across the 19 speakers and then placed onto a treble clef for visualization, with middle G arbitrarily representing the mean habitual pitch of the speakers. Transcriptions were made with Finale PrintMusic 2014.5. Note that this approach presents a picture of the relative pitch – but not the absolute pitch – of the group’s productions, where the absolute pitch was approximately an octave (females) or two (males) lower than what is represented. Virtually all of the single-participant productions fit within the range of a single octave, represented in our transcriptions as a span from middle C to the C one octave above, resulting in roughly equal numbers of semitones in either direction from the G habitual pitch. For the transcriptions presented in **Figures 1**–**7**, only sharps are used to indicate non-diatonic pitches in a C major context. In addition, sharps only apply to the measure they are contained in and do not carry over to the next measure of the transcription.

All of the transcriptions in **Figures 1**–**7** are shown with proposed rhythmic transcriptions in addition to the observed melodic transcriptions. While the purpose of the present study is to quantify speech melody, rather than speech rhythm, the study is in fact a companion to a related one about speech rhythm (Brown et al., 2017). Hence, we took advantage of the insights of that study to present approximate rhythmic transcriptions of the test items in all of the figures. However, it is important to keep in mind that, while the melodic transcriptions represent the actual *results* of the present study, the rhythmic transcriptions are simply approximations generated by the second author and are in no way meant to represent the mean rhythmic trend of the group’s productions as based on timing measurements (as they do in Brown et al., 2017). In other words, the present study was not devoted to integrating our present approach to speech melody with our previous work on speech rhythm, which will be the subject of future analyses.

## Results

The results shown here are the mean pitches relative to each participant’s habitual pitch, where the habitual pitch is represented as middle G on the musical staff. While we are not reporting variability values, we did measure the standard deviation (SD) for each syllable. The mean SD across the 123 syllables in the dataset was 132 cents or 1.32 semitones. For all 19 test items, the last syllable always had the largest SD value. When the last syllable of the test items was removed from consideration, the SD decreased to 120 cents or 1.2 semitones.

### Phrasal Arches

**Figures 1A,B** show the citation forms of two individual words having initial stress, namely yellow (a two-syllable trochee) and telephone (a three-syllable dactyl). As expected for words with initial stress, there is a pitch rise on the first syllable (Lieberman, 1960; Cruttenden, 1997; van der Hulst, 1999), followed by a downtrend of either two semitones (yellow) or three semitones (telephone). **Figure 1C** shows the union of these two words to form the adjectival phrase “The yellow telephone.” Contrary to a prediction based on a simple concatenation of the citation forms of the two words (i.e., two sequential downtrends), there is instead a *contour reversal* for yellow such that there is now a one-semitone rise in pitch between the two syllables (red box in **Figure 1C**), rather than a two-semitone drop. “Telephone” shows a slight compression of its pitch range compared to citation form, but no contour reversal. The end result of this concatenation to form an adjectival phrase is a *melodic arch* pattern (shown by the curved line above the staff in **Figure 1C**), with the pitch peak occurring, paradoxically, on the unstressed syllable of yellow. The initial and final pitches of the phrase are nearly the same as those of the two words in citation form.

**Figures 2A,B** show a similar situation, this time with the initial word having three syllables and the second word having two syllables. As in **Figure 1**, the citation forms of the words show the expected downtrends, three semitones for Saturday and two semitones for morning. Similar to **Figure 1**, the joining of the two words to form a phrase results in a contour change, this time a flattening of the pitches for Saturday (**Figure 2C**, red box), rather than the three-semitone drop seen in citation form. A similar type of melodic arch is seen here as for “The yellow telephone.” As with that phrase, the initial and final pitches of the phrase are nearly the same as those of the two contributing words in citation form. “Morning” shows a small compression, as was seen for “telephone” in **Figure 1C**.

**Figure 3** presents one more example of the comparison between citation form and phrasal concatenation, this time where the word of interest does not have initial stress: the proper name Alanna (an amphibrach foot). **Figure 3A** demonstrates that, contrary to expectations, there is not a pitch rise on the second (stressed) syllable of the word, but that the syllable was spoken with the identical pitch as the first syllable. This is followed by a two-semitone downtrend toward the last syllable of the word. Adding words to create the sentence “Alanna picked it up” again produces a contour reversal to create a melodic arch centered on the unstressed terminal syllable of Alanna (**Figure 3B**, red box).

### Sentence Arches

**Figure 4** picks up where **Figure 1** left off. **Figure 4A** recopies the melody of the phrase “The yellow telephone” from **Figure 1C**. The next two items create successively longer sentences by adding words to the end, first adding the word “rang” and then adding the word “frequently” to the latter sentence. **Figure 4B** shows that the downtrend on “telephone” that occurred when “telephone” was the last word of the utterance is minimized. Instead, there is a *suspension of declination* by a semitone (with reference to the absolute pitch, even though the interval between “le” and “phone” is the same in relative terms). The downtrend then gets shifted to the last word of the sentence, where a terminal drop of a semitone is seen. **Figure 4C** shows a similar phenomenon, except that the word “rang” is part of the suspended declination. The downtrend in absolute pitch now occurs on “frequently,” ending the sentence slightly below the version ending in “rang.” Overall, we see a serial process of suspension of declination as the sentence gets lengthened. One observation that can be gleaned from this series of sentences is that the longer the sentence, the lower the terminal pitch, suggesting that longer sentences tend to have a larger pitch range than shorter sentences. This is also shown by the fact that “yellow” attains a higher pitch in this sentence than in the shorter sentences, resulting in an overall range of five semitones, compared to three semitones for “the yellow telephone.” Hence, for longer sentences, expansions occur at both ends of the pitch range, not just at the bottom.

**Figure 5** compounds the issue of sentence length by now examining sentences with two distinct intonational phrases, each sentence with a main clause and a subordinate clause. The transcriptions now contain two melodic arches, one for each intonational phrase. For illustrative purposes, the phrases of these sentences were all designed to contain components that are found in **Figures 1**–**4**. For the first sentence (**Figure 5A**), the same suspension of declination occurs on the word “rang” as was seen in **Figure 4C**. That this is indeed a suspension process is demonstrated by the fact that the second intonational phrase (the subordinate clause) starts on the last pitch of the first one. The second phrase shows a similar melody to that same sentence in isolation (**Figure 3B**), but the overall pattern is shifted about two semitones downward and the pitch range is compressed, reflecting the general process of declination. Finally, as with the previous analyses, contour reversals are seen with both “yellow” and “Alanna” compared to their citation forms to create melodic arches.

A very similar set of melodic mechanisms is seen for the second sentence (**Figure 5B**). A suspension of declination occurs on “morning” (compared to its phrasal form in **Figure 2C**), and the second intonational phrase starts just below the pitch of “morning.” The phrase “On Saturday morning” shows an increase in pitch height compared to its stand-alone version (**Figure 2B**). In the latter, the pitches for Saturday are three unison pitches, whereas in the longer sentence, the pitches for Saturday rise two semitones, essentially creating an expansion of the pitch range for the sentence. This suggests that longer sentences map out larger segments of pitch space than shorter sentences and that speakers are able to plan ahead by creating the necessary pitch range when a long utterance is anticipated. The second phrase, “the yellow telephone rang,” has a similar, though compressed, intervallic structure compared to when it was a stand-alone sentence (**Figure 4B**), indicating declination effects. In addition, the phrase occurs lower in the pitch range (1–2 semitones) compared to both the stand-alone version and its occurrence in the first phrase of **Figure 5A**, as can be seen by the fact that the transition from “phone” to “rang” is G to F# in the first sentence and F to E in the second. Overall, for long sentences consisting of two intonational phrases, the melody of the first phrase seems to be located in a higher pitch range and shows larger pitch excursions compared to the second intonational phrase, which is both lower in range and compressed in interval size. In other words, more melodic movement happens in the first phrase. As was seen for the set of sentences in **Figure 4**, expansions in pitch range for longer sentences occur at both ends of the range, not just at the bottom.

### Narrow Focus

**Figure 6** examines the phenomenon of narrow focus, where a given word in the sentence is accented in order to place emphasis on its information content. Importantly, the same string of words is found in all four sentences in the figure. All that differs is the locus of narrow focus, which was indicated to participants using block letters for the word in the stimulus sentences. Words under focus are well-known to have pitch rises, and this phenomenon is seen in all four sentences, where a pitch rise is clearly visible on the word under focus, and more specifically its stressed syllable in the case of polysyllabic words “roommate” and “telephone.” All sentences showed terminal drops between “le” and “phones,” although this drop was largest in the last sentence, where the pitch rise occurred on “telephone” and thereby led to an unusual maintenance of high pitch at the end of a sentence. Perhaps the major point to be taken from the results in **Figure 6** is that each narrow-focus variant of the identical string of words had a different melody. Another interesting effect is the contour inversion for “roommate” that occurs when this word precedes the pitch accent (the 1-semitone rise in **Figures 6C,D**), compared to when it follows it (**Figure 6A**) or is part of it (**Figure 6B**). This suggests that, in the former cases, speakers maintain their pitch in the high range in preparation for an impending pitch accent later in the sentence.

### Sentence Modality

**Figure 7** looks beyond declaratives to examine both an imperative statement and two types of interrogatives, namely a yes–no and a WH question (where WH stands for question-words like what, where, and who). **Figure 7A** presents a basic command: “Telephone my house!”. The sentence shows a compressed pitch pattern at a relatively high part of the range, but with a small melodic arch to it, perhaps indicative of the high emotional intensity of an imperative. One noticeable feature here is the loss of the terminal drop that is characteristic of declarative sentences and even citation forms. Instead, pitch is maintained at one general level, making this the most monotonic utterance in the dataset. Perhaps the only surprising result is that the initial stressed syllable “Te” has a slightly lower pitch than the following syllable “le” (79 cents in the raw group data), whereas we might have predicted a slightly higher pitch for the first syllable of a dactyl, as seen in the citation form of “telephone” in **Figure 1B**. Hence, a small degree of arching is seen with this imperative sentence. This stands in contrast to when the first word of a sentence is under narrow focus, as in **Figure 6A** (“MY roommate has three telephones”), where that first word clearly shows a pitch rise.

**Figures 7B,C** present a comparison between the two basic types of questions. The results in **Figure 7B** conform with the predicted pattern of a yes–no question in English, with its large pitch rise at the end (Bolinger, 1989; Ladd et al., 1999; Ladd, 2008; Féry, 2017). The terminal rise of 4 semitones is one of the largest seen in the dataset. The melodic pattern preceding the terminal rise is nearly flat, hence directing all of the melodic motion to the large rise itself. Two features of this sentence are interesting to note. First, whereas long declarative sentences tend to end about three semitones below the habitual pitch, the yes–no question ended a comparable number of semitones above the habitual pitch. Hence, the combination of a declarative sentence and a yes–no interrogative map out the functional pitch range of emotionally neutral speech, which is approximately eight semitones or the interval of a minor 6th. Second, the melodic pattern for “telephone” during the terminal rise is opposite to that in citation form (**Figure 1B**). Next, **Figure 7C** presents the pattern for the WH question “Whose telephone is that?”. The melody is nearly opposite in form to the yes–no question, showing a declining pattern much closer to a declarative sentence, although it lacks the arches seen in declaratives. In this regard, it is closer to the pattern seen with the imperative, although with a larger pitch range and a declining contour. Potential variability in the intonation of this question is discussed in the “Limitations” section below. Overall, the yes–no question and WH-question show strikingly different melodies, as visualized here with notation.

### Interval Use

**FIGURE 8:**
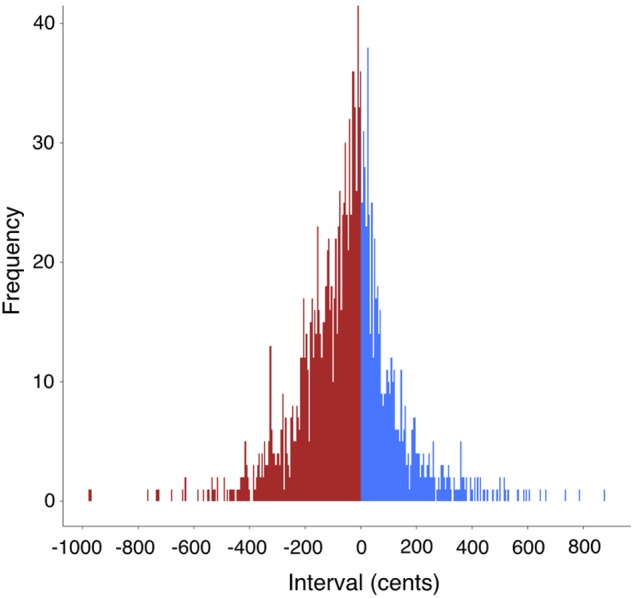
Frequency distribution of interval use in the test corpus. This figure presents the relative frequency of pitch-intervals across the 19 test items and the 19 participants. The *y*-axis represents the absolute frequency of each interval from a pool of 1700 intervals. Along the *x*-axis are the intervals expressed as cents changes, where 100 cents is one equal-tempered semitone. Descending intervals are shown in red on the left side, and ascending intervals are shown in blue on the right side, where the center of the distribution is the unison interval having no pitch change (i.e., two repeated pitches), which was color coded as blue. 96% of the intervals occur in the span of –400 to +400 cents.

**Figure 8** looks at the occurrence of interval categories across all productions of the 19 test-items by the 19 participants. A total of 1700 intervals was measured after discarding items having creaky voice on the terminal syllable. Among the intervals, 37% were ascending intervals (0 cents is included in this group), while 63% were descending intervals. The mean interval size was -45 cents. Fully 96% of the intervals sit in the range of -400 to +400 cents. In other words, the majority of intervals are between a descending major third and an ascending major third, spanning a range of eight semitones or a minor 6th. The figure shows that speech involves small intervallic movements, predominantly unisons, semitones and whole tones, or microtonal intervals in between them. A look back at the transcriptions shows that speech is quite chromatic (on the assumption that our approximation of intervals to the nearest semitone is valid). It is important to point out that the continuous nature of the distribution of spoken intervals shown in **Figure 8** is quite similar to the continuous nature of *sung* intervals for the singing of “Happy Birthday” found in Pfordresher and Brown (2017). Hence, spoken intervals appear to be no less discrete than sung intervals.

Large intervals are rare. They were only seen in situations of narrow focus (**Figure 6**) and the yes–no interrogative (**Figure 7B**), both cases of which were ascending intervals. Large descending intervals were quite rare. A look at the ranges of the sentences across the figures shows that the longest sentences had the largest ranges. Expansion of the range occurred at both the high and low ends, rather than simply involving a deeper declination all on its own, suggestive of phonatory planning by speakers. However, even the longest sentences sat comfortably within the span of about a perfect fifth (seven semitones), with roughly equal sub-ranges on either side of the habitual pitch.

It is difficult to address the question of whether there are scales in speech, since even our longest sentences had no more than 15 pitches, and the constituent intonational phrases had only about 8 pitches. If scaling is defined by the recurrence of pitch classes across a melody, then the overall declination pattern that characterizes the melody of speech does not favor the use of scales. If nothing else, there seems to be a coarse type of chromaticism to the pitch pattern of speech, with semitones (or related microtonal variants) being the predominant interval type beyond the unison. Our working hypothesis is that scaling is a domain-specific feature of music, and that speech is basically an *atonal* phenomenon by comparison, which makes use of a weak type of chromaticism, operating within the compressed pitch range of standard speech production.

## Discussion

We have presented an analysis of speech melody that differs from all contemporary approaches in linguistics but that has similarities to Joshua Steele’s 1775 attempt to capture the melody of speech using symbols similar to musical notation on a musical staff. Compared to other current approaches that merely indicate points of salience or transition in the speech signal, our method permits a quantification of all of the relevant pitch events in a sentence, and does so in a manner that allows for both comparison among speakers and group averaging. This permits a global perspective on speech melody, in addition to simply considering pitch changes between adjacent syllables/tones. We have used this method to analyze a number of key phonetic and phonological phenomena, such as individual words, intonational phrases, narrow focus, and modality. In all cases, the results have provided quantitative insight into these phenomena in a manner that approaches using qualitative graphic markers like H(igh) and L(ow) are unable to.

The general method that we are presenting here consists of three major components: (1) a method for transcribing and thus visualizing speech melody, ultimately uniting melody and rhythm; (2) use of the transcriptions to analyze the structural dynamics of speech melody in terms of intervallic changes and overall pitch movement; and (3) a higher-level interpretation of the pitch dynamics in terms of the phonological meaning of intonation as well as potential comparisons between language and music (e.g., scales, shared prosodic mechanisms). Having used **Figures 1**–**7** to demonstrate the visualization capability of musical transcription, we will now proceed to discuss the results in terms of the dynamics of speech melody.

### Some Melodic Dynamics of Speech

**FIGURE 9:**
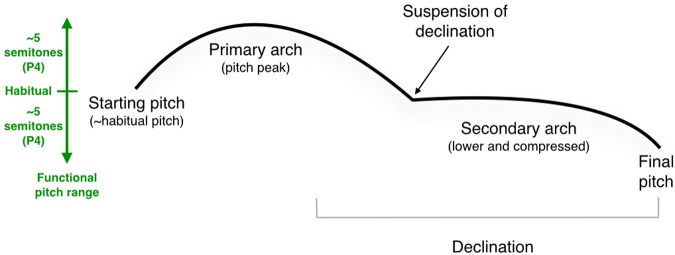
Generic model of the melody of a long declarative sentence. The left side of the figure shows the approximate pitch range for the emotionally neutral intonation of a standard speaker, with the habitual pitch marked in the center, and the functional pitch range mapped out as pitch space above and below the habitual pitch. See text for details about the mechanisms shown. P4, perfect 4th.

**Figure 9** attempts to summarize the major findings of the study by consolidating the results into a generic model of sentence melody for a long declarative sentence containing two principal intonational phrases (as in **Figure 5**). Before looking at the full sentences in the corpus, we first consider the citation form of the individual polysyllabic words that were analyzed. All of them showed the expected phenomenon of a pitch rise on the stressed syllable. This was seen with the words yellow, telephone, Saturday, and morning in **Figures 1**–**4**, but only minimally with Alanna, which showed a pitch drop on the last syllable but not a pitch rise on the stressed syllable.

Looking now to the melodic dynamics of phrases and full sentences, we noted a number of reproducible features across the corpus of test items, as summarized graphically in **Figure 9**.

(1) The *starting pitch* of a sentence tended to be mid-register, at or near a person’s habitual vocal pitch (represented in our transcriptions as middle G). An analysis of the pitch-range data revealed that the habitual pitch was, on average, five semitones or a perfect 4th above a person’s lowest pitch.

(2) Sentences demonstrated an overall *declination* pattern, ending as much as four semitones below the starting pitch, in other words very close to participants’ low pitch. Much previous work has demonstrated declination of this type for English intonation (Lieberman et al., 1985; Ladd et al., 1986; Yuan and Liberman, 2014). The exception in our dataset was the yes–no interrogative, which instead ended at a comparable number of semitones above the habitual pitch. The combination of a declarative and a yes–no interrogative essentially mapped out the functional pitch range of the speakers’ productions in the dataset.

(3) That *pitch range* tended to span about 4–5 semitones in either direction from the habitual pitch for the emotionally neutral prosody employed in the study, hence close to an octave range overall.

(4) Longer sentences tended to occupy a larger pitch range than single words or shorter phrases. The expansion occurred at both ends of the pitch range, rather than concentrating all of the expansion as a greater lowering of the final pitch.

(5) Sentences tended to be composed of one or more *melodic arches*, corresponding more or less to intonational phrases.

(6) Paradoxically, the peak pitch of such arches often corresponded with an unstressed syllable of a polysyllabic word, typically the pitch that followed the stressed syllable.

(7) This was due to the *contour reversal* that occurred for these words when they formed melodic arches, as compared to the citation form of these same words, which showed the expected pitch rise on the stressed syllable.

(8) The *pitch peak* of the arch was quantified intervallically as spanning anywhere from 1 to 3 semitones above the starting pitch of the sentence.

(9) However, melodic arches and other types of pitch accents (like narrow focus) underwent both a *pitch lowering* and *compression* when they occurred later in the sentence, such as in the second intonational phrase of a multi-phrase sentence. In other words, such stress points showed lower absolute pitches and smaller pitch excursions compared to similar phenomena occurring early in the sentence. Overall, for long sentences consisting of two intonational phrases, the melodic contour of the first phrase tended to be located in a higher part of the pitch range and showed larger pitch excursions compared to the second intonational phrase, which was both lower and compressed.

(10) For sentences with two intonational phrases, there was a *suspension of declination* at the end of the first phrase, such that it tended to end at or near the habitual pitch. This suggests that speakers were able to plan out long sentences at the physiological level and thereby create a suitable pitch range for the production of the long utterance. It also suggests that the declarative statement is a holistic formula, such that changes in sentence length aim to preserve the overall contour of the formula.

(11) Sentences tended to end with a small *terminal drop*, on the order of a semitone or two. The exceptions were the imperative, which lacked a terminal drop, and the yes–no interrogative, which instead ended with a large *terminal rise*.

(12) The *terminal pitch* tended to be the lowest pitch of a sentence, underlining the general process of declination. Again, the major exception was the yes–no interrogative.

(13) For declarative sentences, there was a general pattern such that large ascending intervals occurred early in the sentence (the primary melodic arch, **Figure 9**), whereas the remainder of the sentence showed a general process of chromatic descent. This conforms with an overarching driving mechanism of declination.

(14) The overall pitch range tended to be larger in longer sentences, and the terminal pitches tended to be lower as well, by comparison to single words or short phrases.

(15) Speech seems to be dominated by the use of *small melodic intervals*, and hence pitch proximity. Unisons were the predominant interval type, followed by semitones and whole tones, a picture strikingly similar to melodic motion in music (Vos and Troost, 1989; Huron, 2006; Patel, 2008).

(16) Our data showed no evidence for the use of recurrent scale patterns in speech. Instead, the strong presence of semitones in the pitch distribution suggested that a fair degree of chromaticism occurs in speech. Hence, speech appears to be atonal.

### Interpreting the Results in Light of Linguistic Models of Speech Melody

Having summarized the findings of the study according to the musical approach, we would like to consider standard linguistic interpretations of the same phenomena.

#### Phrasal Arches

**FIGURE 10:**
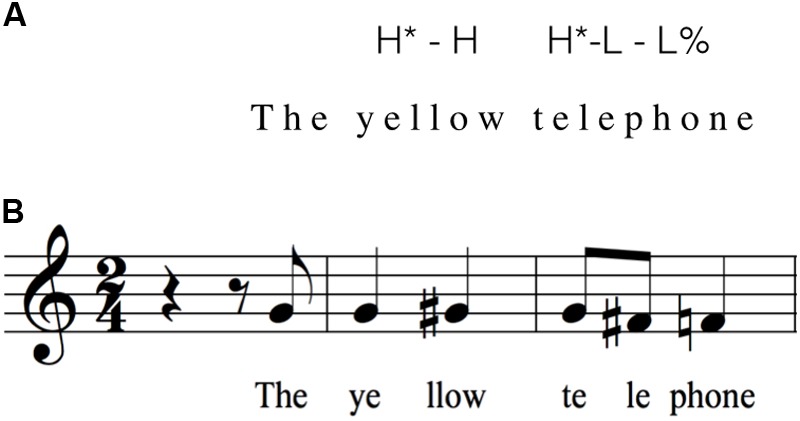
Comparing ToBI transcription and musical transcription. The figure presents a comparison between a ToBI transcription **(A)** and a musical transcription **(B)** of “The yellow telephone.” **(B)** Is a reproduction of **Figure 1C**.

When pronounced in isolation, the stressed syllables of polysyllabic words such as “yellow” and “Saturday” were aligned with a high pitch. The melodic contour then dropped two semitones for the second syllable, resembling that of an utterance-final drop. On the other hand, when “yellow” and “Saturday” were followed by additional words to form short phrases, the melodic contour seen in citation form was inverted, resulting in pitch peaks on the *unstressed* syllables of these words. **Figure 10** presents a direct comparison between a ToBI transcription and a musical transcription for the yellow telephone. AM theory postulates that the pitch-drop in the citation forms of “yellow” and “telephone” represents the transition between the pitch accent (on the stressed syllable) and the boundary tone. In “The yellow telephone,” the (1-semitone) rise would be treated as a transition between the first H^∗^ pitch accent on “yel-” and the H of the H-L-L% boundary tone. But this rise is never treated as a salient phonological event. This change motivates AM theory to consider the “H^∗^-L-L%” tune as compositional, which can be associated with utterances of different lengths (Beckman and Pierrehumbert, 1986). Nonetheless, it is not clear as to why H^∗^ entails a 1-semitone rise, whereas H-L% is manifested by a two semitone drop.

#### Sentence Arches

The observed phrase-level arches – with their contour reversals on polysyllabic words – ultimately lead to the formation of sentence arches in longer sentences. The results of this study indicate that, in general, the melodic contours of utterances consisting of simple subject–verb–object sentences tend to be characterized by a series of melodic arches of successively decreasing pitch height and pitch range. Paradoxically, these arches very often peaked at a non-nuclear syllable, as mentioned above. Additional arches were formed as the sentence was lengthened by the addition of more words or intonational phrases. Moreover, declination was “suspended” when additional syllables were inserted between the pitch accent and the boundary tone (**Figure 4**) or when intonational phrases were added as additional clauses to the sentence (**Figure 5**). To the best of our knowledge, no linguistic theory of speech melody accounts for this suspension. In addition, speakers adjust their pitch range at both ends when producing a long utterance consisting of two intonational phrases. Again, as far as we know, the ability to represent pitch-range changes across a sentence is unique to our model. With both phrases and the boundary tone sharing the same pitch range, the pitch range occupied by each phrase becomes narrower. The first phrase starts at a higher pitch than normal and occupies the higher half of the shared pitch range, while the second phrase begins at a lower pitch and occupies the lower half of the pitch range. At the end of the second phrase, the phrase-final drop is reduced.

To a first approximation, our melodic arches map onto the intonational phrases of phonological theory, suggesting that these arches constitute a key building block of speech melody. For standard declarative sentences, the arches show a progressive lowering in absolute pitch and a narrowing in relative pitch over the course of the sentence, reflecting the global process of declination. Melodic contours of English sentences have been characterized as consisting of different components when comparing the British school with AM theory (Cruttenden, 1997; Gussenhoven, 2004; Ladd, 2008). Despite this, Collier (1989) and Gussenhoven (1991) described a “hat-shape” melodic pattern for Dutch declarative sentences that might be similar to what we found here for English. Whether we are describing the sentence’s speech melody holistically as an arch or dividing the melodic contour into components, we are essentially describing the same phenomenon.

#### Narrow Focus

Comparing the different readings of “My roommate had three telephones” when narrow focus was placed on “my,” “roommate,” “three,” and “telephones” (see **Figure 6**), the results revealed that the stressed syllable of the word under focus was generally marked by a pitch rise of as much as three semitones, except when it occurred on the last word of a sentence, where this pitch jump was absent. Pitch peaks were aligned to the corresponding segmental locations. Both observations are consistent with current research on narrow focus and pitch-segmental alignment in spoken English (Ladd et al., 1999; Atterer and Ladd, 2004; Dilley et al., 2005; Xu and Xu, 2005; Féry, 2017). Xu and Xu’s (2005) prosodic study of narrow focus in British English indicated that, when a word is placed under narrow focus, the pre-focus part of the sentence remains unchanged. This effect is observed in the initial part of the sentences in **Figures 6C,D**, in which the melody associated with “my roommate had” remains unchanged in the pre-focus position. Secondly, Xu and Xu (2005) and Féry (2017) reported that the word under narrow focus is pronounced with a raised pitch and expanded range, whereas the post-focus part of the sentence is pronounced with a lower pitch and more restricted pitch range. These effects were also observed by comparing the sentences **Figures 6C,D** with those in **Figures 6A,B**. The latter part of the sentence “had three telephones” was pronounced in a lower and more compressed part of the pitch range when it is in the post-focus position. Overall, the use of a musical approach to describe narrow focus not only allows us to observe previously reported effects on the pre-, in-, and post-focus parts of the sentence, but it provides a means of quantifying these effects in terms of pitch changes.

#### Sentence Modality

Research in intonational phonology in English indicates that imperative and declarative sentences, as well as WH-questions, are generally associated with a falling melodic contour, whereas the correspondence between speech melody and yes–no (“polar”) questions is less straightforward. Yes–no questions with syntactic inversion (e.g., “Are you hungry?”) are generally associated with a falling melodic contour, whereas those without inversion (e.g., “You are hungry?”) are associated with a rising contour (Crystal, 1976; Geluykens, 1988; Selkirk, 1995). In addition, questions typically involve some element of high pitch (Lindsey, 1985; Bolinger, 1989), whereas such features are absent in statements. While our results are in line with these observations, the comparison of statement and question contours using melodic notation allows us to pinpoint the exact amplitude of the final rises and falls associated with each type of question. Furthermore, it allows us to represent and quantify the difference in global pitch height associated with questions as opposed to statements. This phonologically salient feature is missing in AM and CR, which only account for localized pitch excursions.

### Advantages of a Musical Approach Over Contemporary Linguistic Approaches

The Introduction presented a detailed analysis of the dominant approaches to speech melody in the field of phonology. We would now like to consider the advantages that a musical approach offers over those linguistic approaches.

#### Use of Acoustic Data

Many analyses of speech melody in the literature are based on qualitative representations that show general trajectories of pitch movement in sentences (e.g., Cruttenden, 1997). While useful as heuristics, such representations are inherently limited in scope. Our method is based first and foremost on the acoustic production of sentences by speakers. Hence, it is based on quantitative experimental data, rather than qualitative representations.

#### Quantification and Specification of the Melodic Intervals and Pitch Ranges in Speech

This is in contrast to the use of qualitative labels like H and L in ToBI transcriptions. The musical approach quantifies and thus characterizes the diversity of manners of melodic movement in speech in order to elucidate the dynamics of speech melody. In ToBI, an H label suggests a relative rise in pitch compared to preceding syllables, but that rise is impossible to quantify with a single symbol. The conversion of pitch changes into musical intervals permits a precise specification of the types of pitch movements that occur in speech. This includes both local effects (e.g., syllabic stress, narrow focus, terminal drop) and global effects (e.g., register use, size of a pitch range, melodic arches, intonational phrases, changes with emotion). Ultimately, this approach can elucidate the melodic dynamics of speech prosody, both affective prosody and linguistic prosody.

#### Analysis of All Syllables in an Utterance

This is again in contrast to methods like ToBI that only mark salient pitch events and ignore the remainder of the syllables. Hence, the musical method can provide a comprehensive analysis of the pitch properties of spoken sentences, including the melodic phenomena analyzed here, such pitch-range changes, post-focus compression, lexical stress, narrow focus, sentence modality, and the like. This is a feature which the musical model shares with PENTA.

#### Relative Pitch as a Normalization Procedure for Cross-Speaker Comparison

The use of relative pitch to analyze melodic intervals provides a means of normalizing the acoustic signal and comparing melodic motion across speakers. Hence, normalization can be done across genders (i.e., different registers) and across people having different vocal ranges. In fact, any two individual speakers can be compared using this method. Using relative pitch eliminates many of the problems associated with analyzing speech melody using absolute pitch in Hz. No contemporary approach to speech melody in linguistics provides a reliable method of cross-speaker comparison.

#### Group Averaging of Production

Along the lines of the last point, converting Hz values into cents or semitones opens the door to group averaging of production. Averaging is much less feasible using Hz due to differences in pitch range, for example between women and men. Group averaging using cents increases the statistical power and generalizability of the experimental data compared to methods that use Hz as their primary measurement.

#### Characterizing Variability in Production

A transcriptional approach can be used to capture pitch-based variability in production, as might be associated with regional dialects, foreign accents, or even speech pathology (e.g., hotspots for stuttering, Karniol, 1995). As we will argue in the Limitations section below, it can also capture the variability in the intonation of a single sentence across speakers, much as our analysis of narrow focus did in **Figure 6**, showing that each variant was a accompanied by a distinct melody.

#### A Unification of Melody and Rhythm

Virtually all approaches to speech prosody look at either melody or rhythm alone. Following on the landmark work of Joshua Steele in 1775, we believe that our use of musical notation provides an opportunity for such a unification. We have developed a musical model of speech rhythm elsewhere (Brown et al., 2017). That model focuses on meters and the relative duration values of syllables within a sentence. We used approximate rhythmic transcriptions in the current article (**Figures 1**–**7**) to demonstrate the potential of employing a combined analysis of melody and rhythm in the study of speech prosody. We hope to do a combined rhythm/melody study as the next phase of our work on the musical analysis of speech.

As mentioned in the Introduction, RaP (Rhythm and Pitch) is perhaps the one linguistic approach that takes into account both speech rhythm and melody, albeit as completely separate parameters (Dilley and Brown, 2005; Breen et al., 2012). RaP considers utterances as having “rhythm,” which refers to pockets of isochronous units in lengthy strings of syllables (at least 4–5 syllables, and up to 8–10 syllables). In addition, “strong beats” associate with lexically stressed syllables based on metrical phonology. RaP is the first recent model to make reference to the musical element of “beat” in describing speech rhythm, implying that some isochronous units of rhythm exist at the perceptual level. However, the assignment of rhythm and prominence relies heavily on transcribers’ own perception, rather than on empirical data.

#### Speech/Music Comparisons

The use of musical notation for speech provides a means of effecting comparative analyses of speech and music. For example, we explored the question of whether speech employs musical scales, and concluded provisionally that it does not. There are many other types of questions about the relationship between speech prosody and music that can be explored using transcription and musical notation. This is important given the strong interest in evolutionary models that relate speech and music (Brown, 2000, 2017; Mithen, 2005; Fitch, 2010), as well as cognitive and neuroscientific models that show the use of overlapping resources for both functions (Juslin and Laukka, 2003; Besson et al., 2007; Patel, 2008; Brandt et al., 2012; Bidelman et al., 2013; Heffner and Slevc, 2015). For example, it would be interesting to apply our analysis method to a tone language and attempt to quantify the production of lexical tones in speech, since lexical tone is thought of as a relative-pitch system comprised of contrastive level tones and/or contour tones.

#### The Score Allows a Person’s Intonation to Be Producible by Someone Else

The use of a musical score is the only visual method that can allow a person to reproduce the prosody of some other person. Hence, the score can be “sung” much the way that music is. While this is certainly an approximation of the pitch properties of real speech, it is unquestionably a huge improvement over any existing method in linguistics, including Prosogram. A system integrating speech rhythm and melody could enable the development of more-effective pedagogical tools to teach intonation to non-native language learners. Moreover, knowledge gleaned from this research can be applied to improve the quality and naturalness of synthesized speech.

### Limitations

In addition to the advantages of the musical approach, there are also a number of limitations of our study and its methods. First, we used a simple corpus with relatively simple sentences. We are currently analyzing a second dataset that contains longer and more complex sentences than the ones used in the present study. These include sentences with internal clauses, for example. Second, our pitch analysis is very approximate and is no more fine-grained than the level of the semitone. All of our analyses rounded the produced intervals to the nearest semitone. If speech uses microtonal intervals and scales, then our method at present is unable to detect them. Likewise, our association of every syllable with a level tone almost certainly downplays the use of contour tones (glides) in speech. Hence, while level tones should be quite analyzable with our method, our approach does not currently address the issue of intra-syllable pitch variability, which would be important for analyzing contour tones in languages like Mandarin and Cantonese. Prosogram permits syllabic pitches to be contoured, rather than level, but our approach currently errs on the side of leveling out syllabic pitches. In principle, contour tones could be represented as melismas in musical notation by representing the two pitches that make up the endpoints of the syllable and using a “portamento” (glide) symbol to suggest the continuity of pitch between those endpoints. A similar approach could even be used to represent non-linguistic affective vocalizations.

The current approach requires that users be familiar with musical notation and the concept of musical intervals. Will this limit the adoptability of the approach? In our opinion, it is not much more difficult to learn how to read musical notation than it is to learn how to read ToBI notation, with its asterisks and percentage signs. In principle, pitch contours should be easily recognizable in musical notation, even for people who cannot read it. Hence, the direction and size of intervals should be easy to detect, since musical notation occurs along a simple vertical grid, and pitch changes are recognizable as vertical movements, much like lines representing F_0_ changes. In contrast to this ease of recognition, ToBI notation can be complex. The fact that H^∗^H means a flat tone is completely non-intuitive for people not trained in ToBI. The most “musical” part of musical notation relates to the interval classes themselves. This type of quantification of pitch movement is not codable at all with ToBI and thus represents a novel feature that is contributed by musical notation.

Our sample showed a gender bias in that 16 of the 19 participants were female. The literature suggests that females show greater *F*_0_ variability than males (Puts et al., 2011; Pisanski et al., 2016) and that they have a higher incidence of creaky voice (Yuasa, 2010). Creaky voice was, in fact, a problem in our analysis, and this might have been well due to the high proportion of females in our sample. Future studies should aim to have a more balanced gender representation than we were able to achieve in this study.

**FIGURE 11:**
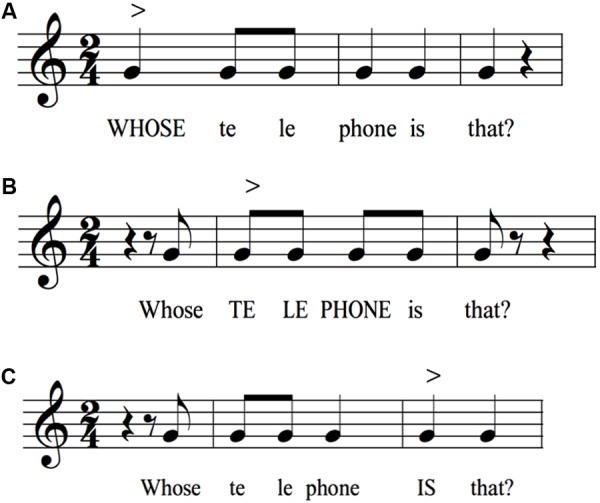
Variability in the intonation of a single sentence. The figure uses rhythmic notation to demonstrate three possible ways of intoning the same string of words, where stress is placed on either whose **(A)**, telephone **(B)**, or is **(C)**. It is expected that different melodic patterns would be associated with each rendition, based on where the point of focus is, which would be an attractor for a pitch rise. The proposed point of narrow focus is represented using bold text in each sentence. The symbol “>” signifies a point of focus or accent. The pattern in **(A)** was most consistent with the analyzed group-average melody shown in **Figure 7C**.

Finally, while our normalization of the speech signal into semitones provides a strong advantage in that it permits group averaging, such averaging also comes at the cost of downplaying individual-level variability. Perhaps instead of averaging, it would be better to look at the families of melodies for a single sentence that is produced by a group of speakers, and put more focus on the individual-level variability than on group trends. In order to illustrate the multiple ways that a single sentence can be intoned, we revisit the WH-question that was analyzed in **Figure 7C**: “Whose telephone is that?”. **Figure 11** uses rhythmic transcription to demonstrate three different manners of intoning this question, the first of which was used in **Figure 7C** (for simplicity, a single G pitch is used in all transcriptions). Each variant differs based on where the point of focus is, as shown by the word in block letters in each transcription. We chose the version in **Figure 10A** for our group analysis in **Figure 7C**, since the melodic pattern of the group average best fit that pattern, with its high pitch on “whose,” rather than on “telephone” or “is.” Hence, while the examination of group averages might tend to downplay inter-participant variability, the transcriptional approach is able to capture the family of possible variants for a given sentence and use them as candidates for the productions of individuals and groups.

## Conclusion

The musical method that we are presenting here consists of three major components: (1) a method for transcribing and thus visualizing speech melody, ultimately uniting melody and rhythm into a single system of notation; (2) use of these transcriptions to analyze the structural dynamics of speech melody in terms of intervallic changes and pitch excursions; and (3) a higher-level interpretation of the descriptive pitch dynamics in terms of the phonological meaning of intonation as well as potential comparisons between speech and music (e.g., scales, shared prosodic mechanisms). Application of this approach to our vocal-production experiment with 19 speakers permitted us to carry out a quantitative analysis of speech melody so as to look at how syntax, utterance length, narrow focus, declination, and sentence modality affected the melody of utterances. The dominant linguistic models of speech melody are incapable of accounting for such effects in a quantifiable manner, whereas such melodic changes can be easily analyzed and represented with a musical analysis. This can be done in a comprehensive manner such that all syllables are incorporated into the melodic model of a sentence. Most importantly, the use of a musical score has the potential to combine speech melody and rhythm into a unified representation of speech prosody, much as Joshua Steele envisioned in 1775 with his use of “peculiar symbols” to represent syllabic pitches. Musical notation provides the only available tool capable of bringing about this unification.

## Author Contributions

IC and SB analyzed the acoustic data and wrote the manuscript.

## Conflict of Interest Statement

The authors declare that the research was conducted in the absence of any commercial or financial relationships that could be construed as a potential conflict of interest.
